# Brain imaging features in schizophrenia with co‐occurring auditory verbal hallucinations and depressive symptoms—Implication for novel therapeutic strategies to alleviate the reciprocal deterioration

**DOI:** 10.1002/brb3.1991

**Published:** 2020-12-11

**Authors:** Chuanjun Zhuo, Tao Fang, Ce Chen, Min Chen, Yun Sun, Xiaoyan Ma, Ranli Li, Hongjun Tian, Jing Ping

**Affiliations:** ^1^ Key Laboratory of Real Time Brain Circuits Tracing of Neurology and Psychiatry (RTBNB_Lab) Tianjin Fourth Center Hospital Tianjin Medical Affiliated Tianjin Fourth Central Hospital Nankai University Affiliated Tianjin Fourth Center Hospital Tianjin China; ^2^ Department of Psychiatry Wenzhou Seventh People’s Hospital Wenzhou China; ^3^ Psychiatric‐Neuroimaging‐Genetics‐Comorbidity (PNGC) Laboratory Tianjin Mental Health Center Tianjin Anding Hospital Nankai University Affiliated Anding Hospital Tianjin China; ^4^ Department of Psychiatry School of Mental Healthy Jining Medical University Jining China

**Keywords:** auditory verbal hallucinations, brain feature, depression, Schizophrenia, tailor treatment

## Abstract

**Background:**

Auditory verbal hallucinations (AVHs) and depressive symptoms are highly prevalent in schizophrenia, and recent progress has been made in understanding the reciprocal deterioration of both symptoms through structural and functional brain imaging studies. To date, there is limited literature on this topic. In this review, we synthesized the recent literature on the neuroimaging features of schizophrenia patients with concurrent AVHs and depressive symptoms.

**Methods:**

A literature search was conducted with the major databases using the keywords, mainly including schizophrenia, AVHs, depression, neuropsychiatric disorders, brain imaging, and magnetic resonance imaging.

**Results:**

The existing studies have shown that AVHs and depressive symptoms reciprocally deteriorate in patients with schizophrenia, which has challenged the conventional treatment of the disease. Interestingly, repetitive transcranial magnetic stimulation (rTMS) and transcranial direct current stimulation (tDCS) therapies have emerged as two efficacious brain stimulation treatments that can normalize the brain regions associated with the symptoms, as shown through functional and structural brain imaging studies. In light of these important findings, there is an urgent need to conduct in‐depth neuronal mechanistic studies to identify targets for stimulation therapy.

**Conclusions:**

These new findings may elucidate the pathological mechanisms underlying schizophrenia with concurrent AVHs and depressive symptoms. Furthermore, this review has important clinical implications for developing novel therapeutic strategies to alleviate the reciprocal deterioration AVHs and depressive symptoms of schizophrenia patients.

## Graphical abstract

1. Auditory verbal hallucinations (AVHs) and depressive symptoms are highly prevalent in schizophrenia. 2. The existing studies have shown that AVHs and depressive symptoms reciprocally deteriorate in patients with schizophrenia, which has posed changes in the conventional treatment of the disease. 3. There is an urgent need for in‐depth neuronal mechanistic studies to identify targets for the development effective therapies to alleviate the reciprocal deterioration.

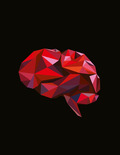

## 1 INTRODUCTION

Auditory verbal hallucinations (AVHs) are a characteristic symptom in schizophrenia, occurring in approximately 70% of patients. Patients with AVHs usually hear unreal “voices” in the absence of any external sound stimulation, according to the International Consortium on Hallucination Research (Waters et al., 2014). Notably, AVHs in schizophrenia are characterized by negative “voices” against the patients themselves or hostile comments, which may be satirizing, insulting, and commanding. As such, AVHs can cause considerable mental distress to patients, especially when they are an initial symptom. Insulting “voices” can lead to self‐harm, suicidal attempts, suicide, and extreme violence in patients with schizophrenia (Dugré et al., 2018). Therefore, there is an urgent need to understand the underlying neural mechanisms to identify therapeutic targets for the effective treatment of AVHs (Fujita et al., 2015; Hugdahl, 2015).

In addition to AVHs, depressive symptoms are common in schizophrenia, with up to 80% of patients with schizophrenia experiencing depressive episodes during the early stages of the disease (Bosanac & Castle, 2012; Fusar‐Poli et al., 2013). Studies over the past 5 years have shown that depressive symptoms play a destructive role in the prognosis of patients with schizophrenia, decreasing the efficacy of treatment (Bosanac & Castle, 2012; Fusar‐Poli et al., 2013). It has also been noted that depressive symptoms can increase the risk of self‐harm and suicide in schizophrenia patients with concurrent depression.

Recent studies have shown that depressive symptoms and AVHs often co‐occur in a proportion of patients with schizophrenia (Krynicki et al., 2018; McGinty & Upthegrove, 2020), and the concurrence greatly increases the risk of self‐harm and suicide (Gardsjord et al., 2016; Kelleher et al., 2013). Studies from the past 5 years have suggested that AVHs and depressive symptoms reciprocally worsen the mental state of patients with schizophrenia, resulting in further aggravation of cognitive impairment and decline of social functioning (Chiang et al., 2018; Helfer et al., 2016; Janaki et al., 2017; Liu et al., 2019; Wang et al., 2019). Due to our long‐standing interest in this topic, we used brain imaging techniques to identify structural and functional brain imaging signatures and to investigate the neural mechanisms that underline AVHs and depressive symptoms in schizophrenia. We found that gray matter (GM) damage was more severe in first‐episode schizophrenia patients with AVHs and depressive symptoms, with reductions in GM volume in the parietal lobe, frontal lobe, and temporal lobe, especially in the Broca and Wernicke areas, and prefrontal lobe. Moreover, we noticed that first‐episode schizophrenia patients with concurrent AVHs and depressive symptoms had an average global GM atrophy of 1.6%, significantly higher than that of 0.58% in patients with depressive symptoms alone. The severe brain damage can make treatment more difficult, leading to the high morbidity and mortality rates associated with the disease (Maximilian et al., 2017). Therefore, there is an urgent need to elucidate the neural mechanisms for the reciprocal deterioration of the concurrent symptoms, as to identify novel therapeutic targets and treatments for schizophrenia (Krynicki et al., 2018; McGinty & Upthegrove, 2020).

In this review, we summarize the recent findings, including the results from our laboratory, on the structural and functional brain imaging characteristics of schizophrenia with concurrent AVHs and depressive symptoms. As the treatment of schizophrenia with concurrent AVHs and depressive symptoms is challenging, the recent findings described in this review have important clinical implications for the development of new therapeutic strategies to alleviate the reciprocal deterioration and improve the treatment of schizophrenia in the clinic, and the main findings are listed in Table 1.

**Table 1:** Summary of the main references according to different categories related to AVHs and depressive symptoms in patients with schizophrenia

Authors	Title	Journal	Published time	Main findings
Structural brain imaging features of AVHs in schizophrenia				
Kubera et al.	Structure/function interrelationships in patients with schizophrenia who have persistent auditory verbal hallucinations: A multimodal MRI study using parallel ICA.	*Neuro Psychopharmacology & Biological Psychiatry*.	2019	Gray matter volume reduction in fronto‐temporoparietal regions.
Modinos et al.	Neuroanatomy of auditory verbal hallucinations in schizophrenia: a quantitative meta‐analysis of voxel‐based morphometry studies.	*Cortex*.	2013	Gray matter disturbance is found in bilateral superior temporal gyri (including Heschl's gyri), which is key areas of structural pathology in AVHs schizophrenia.
Di Biase et al.	Neuroimaging auditory verbal hallucinations in schizophrenia patient and healthy populations.	*Psychol Med*.	2020	White matter disturbance is found in the genu and splenium of the corpus callosum, as well as the anterior limbs of the internal capsule.
Modinos et al.	Neuroanatomy of auditory verbal hallucinations in schizophrenia: a quantitative meta‐analysis of voxel‐based morphometry studies.	*Cortex*.	2013	GM connections between the primary auditory cortex and secondary auditory cortex disturbed.
Psomiades et al.	Integrity of the arcuate fasciculus in patients with schizophrenia with auditory verbal hallucinations: A DTI‐tractography study.	*Neuroimage Clin*.	2016	Arcuate fasciculus tract connecting the auditory cortex and language processing loop is damaged.
Di Biase et al.	Neuroimaging auditory verbal hallucinations in schizophrenia patient and healthy populations.	*Psychol Med*.	2020	Corpus callosum damaged.
Functional brain imaging features of AVHs in schizophrenia				
Alonso‐Solís et al.	Altered amplitude of low frequency fluctuations in schizophrenia patients with persistent auditory verbal hallucinations.	*Schizophr Res*.	2017	Higher ALFFs are found in the bilateral thalamus, parahippocampal gyrus, putamen, insular lobe, and inferior frontal pole.
Cui et al.	Putamen‐related regional and network functional deficits in first‐episode schizophrenia with auditory verbal hallucinations.	*Schizophr Res*.	2016	ReHo increases can be found in the right dorsolateral prefrontal lobe.
Zhuo et al.	Differences in functional connectivity density among subtypes of schizophrenic auditory hallucination.	*Brain Imaging Behav*.	2020	Abnormal functional connection density.
Ćurčić‐Blake et al.	Interaction of language, auditory and memory brain networks in auditory verbal hallucinations.	*Prog Neurobiol*.	2017	Memory unstable hypothesis. Source monitoring hypothesis. Transmission of false information between hemispheres hypothesis. Top‐down effect and bottom‐up predictive dissonance hypothesis. Mixed model hypothesis.
Structural brain imaging features of schizophrenia and depression				
Arnone et al.	Altered amplitude of low frequency fluctuations in schizophrenia patients with persistent auditory verbal hallucinations.	*Schizophr Res*.	2013	GMV atrophy is observed in the prodromal phase of monophasic depression and schizophrenia.
Liu et al.	Impact of emotion dysregulation and cognitive insight on psychotic and depressive symptoms during the early course of schizophrenia spectrum disorders.	*Early Interv Psychiatry*.	2019	GM damage is more severe in first‐episode schizophrenia patients with depressive symptoms than patients without depressive symptoms.
Functional brain imaging features of schizophrenia and depression				
Kumari et al.	Mapping Depression in Schizophrenia: A Functional Magnetic Resonance Imaging Study.	*Schizophr Bull*.	2016	Substantial functional activation of several brain regions is observed in patients with depression and schizophrenia, including the thalamus.
Schilbach et al.	Transdiagnostic commonalities and differences in resting state functional connectivity of the default mode network in schizophrenia and major depression.	*Neuroimage Clin*.	2015	Reduced functional connectivity is found between the anterior cuneiform lobe and bilateral superior parietal lobule in patients with depression and schizophrenia.
Wu et al.	Functional network connectivity alterations in schizophrenia and depression.	*Psychiatry Res Neuroimaging*.	2017	Decreased connectivity of the left control network and medial visual networks, are common functional brain characteristics of schizophrenia and depression.
Han et al.	Low‐rank network signatures in the triple network separate schizophrenia and major depressive disorder.	*Neuroimage Clin*.	2019	Abnormal density of functional connections is found in the prefrontal lobe of patients with depression and schizophrenia.
Wei et al.	Similarities and differences of functional connectivity in drug‐naïve, first‐episode adolescent and young adult with major depressive disorder and schizophrenia.	*Sci Rep*.	2017	Abnormalities of regional functional connectivity, primarily in the bilateral orbitofrontal lobe, is a common brain characteristic of schizophrenia, depression, and bipolar disorder.
Ma et al.	Transdiagnostic Dysfunctions in Brain Modules Across Patients with Schizophrenia, Bipolar Disorder, and Major Depressive Disorder: A Connectome‐Based Study.	*Schizophr Bull*.	2019	Increased connection coefficients are found between certain networks, especially networks of the dorsolateral prefrontal lobe, angular gyrus, and thalamus.
Qi et al.	The relevance of transdiagnostic shared networks to the severity of symptoms and cognitive deficits in schizophrenia: a multimodal brain imaging fusion study.	*Transl Psychiatry*.	2020	FCD is impaired in first‐episode schizophrenia patients with comorbid depression.
Co‐occurring AVHs and depressive symptoms in schizophrenia				
Siddi et al.	Depression, auditory‐verbal hallucinations, and delusions in patients with schizophrenia: Different patterns of association with prefrontal gray and white matter volume.	*Psychiatry Res Neuroimaging*.	2019	GM volume reductions in the prefrontal lobe are associated with depressive symptoms and AVHs in schizophrenia patients.
Zhuo et al.	Depressive symptoms combined with auditory hallucinations are accompanied with severe gray matter brain impairments in patients with first‐episode untreated schizophrenia ‐ A pilot study in China.	*Neurosci Lett*.	2020	The loss of GM volume in schizophrenia patients with concurrent AVHs and depressive symptoms is significantly more severe than those patients with depressive symptoms alone.
Implications for novel therapeutic strategies to alleviate the reciprocal deterioration of AVHs and depressive symptoms in schizophrenia				
Zöllner et al.	Theta‐Burst Stimulation for Auditory‐Verbal Hallucination in Very‐Late‐Onset Schizophrenia‐Like Psychosis‐A Functional Magnetic Resonance Imaging Case Study.	*Front Psychiatry*.	2020	Modified and applied continuous theta‐burst stimulation alleviates AVHs in patients with schizophrenia.
2019	Neurofeedback of core language network nodes modulates connectivity with the default‐mode network: A double‐blind fMRI neurofeedback study on auditory verbal hallucinations.	*Neuroimage*.	2019	tfMRI effectively alleviates the symptoms of AVHs.
Dellazizzo et al.	Avatar Therapy for Persistent Auditory Verbal Hallucinations in an Ultra‐Resistant Schizophrenia Patient: A Case Report.	*Front Psychiatry*	2018	AVATAR effectively alleviates the symptoms of AVHs.
Summary and future direction				
Waters et al.	Auditory hallucinations in schizophrenia and nonschizophrenia populations: a review and integrated model of cognitive mechanisms.	*Schizophr Bull*.	2012	Orbitofrontal cortex plays an essential role in the interaction between self‐monitoring or social behavior and emotional valence. The adverse effect of depressive episodes on the GM orbitofrontal volume may contribute to the self‐monitoring disturbances, which may result in AVHs orbitofrontal, right medial superior frontal, superior frontal, and middle frontal gyrus.
Beer et al.	Orbitofrontal cortex and social behavior: Integrating self‐monitoring and emotion‐cognition interactions.	*Journal of Cognitive Neuroscience*.	2006	Depressive symptoms during the early stages of schizophrenia may exert a causal role in triggering the development of AVHs.

Note

Normalized the functional disruption of orbitofrontal cortex and orbitofrontal cortex‐related networks may be a new target to alleviate AVHs and depressive symptoms in patients with schizophrenia.

## 2 AVHS IN SCHIZOPHRENIA

### 2.1 Structural brain imaging features of AVHs in schizophrenia

#### 2.1.1 Abnormal reductions in gray matter (GM) volume

Previous studies have shown that AVHs in schizophrenia are associated with several abnormal brain regions, including the parietal lobe, temporal lobe, insular lobe, frontal lobe, and temporoparietal syndesmosis (Kubera et al., 2019). The reductions in GM volume and cortical complexity are also observed in schizophrenia patients with AVHs, with impairments more severe in the left hemisphere than the right hemisphere (Modinos et al., 2013). As the GM‐heavy regions in the brain include important areas of the executive control network, attention network, memory network, and emotional network, and default mode network (Alderson‐Day et al., 2015; Ćurčić‐Blake et al., 2017; Hugdahl, 2017), damaged GM is often associated with the first‐episode and chronic schizophrenia. These previous findings suggest that structural abnormalities, primarily decreased GM volumes, may underlie the occurrence and persistence of AVHs in patients with schizophrenia.

#### 2.1.2 Abnormal reduction in white matter (WM) volume

In addition to GM reductions, previous studies have revealed abnormal reductions in white matter (WM) volumes of schizophrenia patients with AVHs, mainly in the arcuate tract, corpus callosum, superior longitudinal tract, and inferior longitudinal bundle (Alderson‐Day et al., 2015; Catani et al., 2011; Di Biase et al., 2020; Kubera et al., 2019; Modinos et al., 2013; Pomponio et al., 2020). Due to the importance of these fiber bundles in connecting the left and right hemispheres of the brain, along with making connections between the anterior and posterior regions of the brain, these damaged WM‐heavy regions are closely associated with the occurrence and persistence of AVHs in patients with schizophrenia.

#### 2.1.3 Abnormal alterations in the WM and GM junctions

Studies on GM and WM junction techniques have shown that the GM and WM junctions are damaged in patients with AVHs. For example, Modinos et al. (2013) observed a decrease in GM connections between the primary auditory cortex and secondary auditory cortex of patients with AVHs. At the same time, Allen et al., (2012) identified WM junction damage in a top‐down regulatory pathway of the auditory cortex. In another study, Psomiades and colleagues found that the arcuate fasciculus tract connecting the auditory cortex and language processing loop was damaged (Psomiades et al., 2016). Several studies have also detected damage to the corpus callosum of patients with AVHs (Di Biase et al., 2020; Leroux et al., 2017; Xi et al., 2016). A previous study reported that WM fiber bundle damage from the top to bottom and back to front in patients with AVHs (Di Biase et al., 2020). While a few studies have detected increases in the WM anisotropy (FA) of patients with AVHs, these findings are inconsistent with the findings from most previous studies, including ours (Mulert et al., 2012; Shergill et al., 2007; Xie et al., 2019). Due to the conflicting results, studies with large sample sizes are needed to clarify these findings.

### 2.2 Functional brain imaging features of AVHs in schizophrenia

Aberrant brain activity and disrupted functional connectivity (FC) are important neural signatures of schizophrenia. Functional brain abnormalities have been characterized in schizophrenia patients with AVHs using the amplitude of low‐frequency fluctuation (ALFF) technique. ALFF is a proven method with a capacity to precisely reflect the intensity or power of regional spontaneous brain activity, regional homogeneity (ReHO), resting‐state functional connectivity (rsFC), resting‐state global functional connectivity density, effective connectivity (EC) large brain network, and interactions between the subnetworks. Some functional brain abnormalities have also been identified in our previous studies. The recent progress and advancements in the understanding of functional brain imaging features in schizophrenia patients with AVHs are summarized as follows:

Regardless of the presence of AVHs, previous studies have shown that schizophrenia patients have higher ALFFs in the bilateral thalamus, parahippocampal gyrus, putamen, insular lobe, and inferior frontal pole, as compared with healthy controls (Alonso‐Solís et al., 2017). When comparing schizophrenia patients with and without AVHs, schizophrenia patients with AVHs show lower ALFFs in the left putamen. However, ReHo increases can be found in the right dorsolateral prefrontal lobe. When the right putamen was set as the seed region, spontaneous fluctuations of brain activity at rest, also referred to as rsFC, were increased between the right putamen, left dorsolateral prefrontal lobe, and the left Broca area (Cui et al., 2016). In addition, schizophrenia patients with AVHs show aberrant functional connections in the anterior cingulate gyrus, insular lobe, and language‐related brain regions (Alonso‐Solís et al., 2017). Notably, a previous study found that brain network connections were disrupted in schizophrenia patients with first‐episode AVHs (Li et al., 2017). Hence, the EC between the thalamus, auditory cortex, and hippocampus may be a neural feature of AVHs in patients with schizophrenia (Mechelli et al., 2007). In addition, schizophrenia patients with AVHs also show reductions in EC between the middle temporal gyrus and language‐related networks (Zhao et al., 2018). In two recent studies of ours, we found that schizophrenia patients with AVHs had abnormal functional connection density (FCD) (Zhuo, Li, et al., 2020), with different types of AVHs exhibiting specific changes in FCD (Zhuo, Li, et al., 2020).

Due to rapid advances in functional imaging technologies, a variety of algorithms have been developed and widely implemented for functional brain imaging. The networks related to AVHs in patients with schizophrenia primarily include the imbalance of FCs or ECs in the following networks: (a) A network of auditory and language‐related brain regions, mainly including the left inferior frontal gyrus, left superior temporal gyrus, and left middle temporal gyrus; (b) default network‐related brain areas with the lateral temporoparietal junction, midline structure, and hippocampal region; and (c) a network composed of the central executive network, inferior parietal lobule, middle frontal gyrus, posterior cingulate, inferior frontal gyrus, auxiliary motor area, angular gyrus, superior parietal lobule, orbitofrontal lobe, ventral prefrontal lobe, sensory synaptic cortex, and striatum (Alderson‐Day et al., 2015).

Based on the characteristics of FCs between the relevant brain regions, different brain networks, the subcomponents of different brain networks, and brain networks and brain regions, the following hypotheses of AVHs in schizophrenia have been proposed (Ćurčić‐Blake et al., 2017):

1. *Memory unstable hypothesis*. The disrupted memory, such as the fragmentation of memory, may be associated with AVH symptoms, including inner speech and auditory images as parasitic symptoms in language processing. Disorders of the memory coding system cause dysfunctional memory coding, leading to memory disorders and the appearance of parasitic memory fragments that do not match the original environment of the AVHs. Several studies have detected the abnormal transmission of information between the hippocampal–putamen–language and auditory processing networks (i.e., the Broca and Wernicke regions). Based on clinical symptoms, there is a correlation between childhood traumatic experience and AVHs, which supports the unstable memory hypothesis. The results in our studies also support the unstable memory hypothesis of AVHs.
2. *Source monitoring hypothesis*. It has been hypothesized that AVHs are caused by the functional defects between brain self‐monitoring and fact identification. The functional impairments cause problems with “thinking, internal language, and partial spontaneous brain activity,” resulting in self‐cognitive difficulties and AVHs.
3. *Transmission of false information between hemispheres hypothesis*. It has been proposed that the excessive synchronization of the auditory cortex of the left and right hemispheres can lead to AVHs.
4. *Top‐down effect and bottom‐up predictive dissonance hypothesis*. It has also been hypothesized that AVHs may be related to functional imbalances between the bottom‐up transmission of the sensory system and the top‐down processing of information. The bottom‐up information transmission is more inclined to perceptual processing, while the top‐down regulation is more associated with general processing. It is thought that this imbalance may lead to the development of AVHs.
5. *Mixed model hypothesis*. In 2013, Ford and Hoffman proposed the mixed model hypothesis of AVHs, of which cortical striatal disconnection is the neural basis (Ford & Hoffman, 2013). Other mixed model hypotheses have also been created. For example, Waters *et al*. integrated the top‐down regulation imbalance and self‐monitoring ability defects to create a mixed model hypothesis (Aleman & Larøi, 2011; Waters et al., 2012), while Northoff proposed a hypothesis of misconnection between multiple networks (Northoff & Qin, 2014). Although different mixed model hypotheses have been supported by scientific evidence, there have been several limitations.

Therefore, the relationship between the interaction of language–hearing–memory networks and AVHs in schizophrenia needs to be further studied. In particular, an in‐depth investigation of the language network and its connection with the memory and auditory networks need to be performed. In addition, further studies are needed on information transmission and communication dysfunction among the three networks, to further elucidate the neural mechanisms of AVHs.

## 3 DEPRESSIVE SYMPTOMS IN SCHIZOPHRENIA

Compared with the large number of magnetic resonance imaging (MRI)‐based studies on schizophrenia patients with concurrent AVHs, there are relatively fewer brain imaging studies on schizophrenia patients with concurrent depressive symptoms. The brain signature of depressive symptoms in schizophrenia remains to be characterized. The existing studies focused on the common characteristics of brain damage, which is also known as the shared loop, in schizophrenia and depression. The primary findings from these studies are summarized below.

### 3.1 Structural brain imaging features of schizophrenia and depression

Schizophrenia and depression share common characteristic brain structural abnormalities. For example, insular GM volumes are decreased in patients with first‐episode affective disorder and first‐episode schizophrenia, which is progressively aggravated with the progression of the disease (Lee et al., 2016). Recently, Arnone et al. (2013) found similar patterns of hippocampal GMV atrophy in the prodromal phase of monophasic depression and schizophrenia. In our previous study, we observed that GM damage was more severe in first‐episode schizophrenia patients with depressive symptoms than patients without depressive symptoms (Liu et al., 2020).

### 3.2 Functional brain imaging features of schizophrenia and depression

In terms of abnormal brain function, Busatto (2013) found structural and functional alterations of the hippocampus, insular lobe, prefrontal lobe, and other brain regions in depressive patients with psychotic symptoms, similar to the emotional processing loop in patients with schizophrenia. Using task‐based functional imaging techniques, Kumari et al. (2016) found that substantial functional activation of several brain regions in patients with depression and schizophrenia, including the thalamus. Dysfunction of the amygdala is a common phenomenon of the mood‐processing loop, in diseases like affective disorders and schizophrenia (Broome et al., 2015). In another study, Schilbach et al. (2015) detected reduced functional connectivity between the anterior cuneiform lobe and bilateral superior parietal lobule in patients with depression and schizophrenia. In another study, Kumari *et al*. indicated that specific brain signatures of schizophrenia patients with depressive symptoms might “extended from the left thalamus to putamen–globus pallidus–insular lobe–inferior frontal gyrus–middle frontal gyrus–precentral gyrus.” In contrast, other brain regions were activated abnormally under the stimulation of fear cues (Kumari et al., 2016).

In 2017, it was found that positive connectivity in the control network, composed of the left and right parietal lobe, and decreased connectivity of the left control network and medial visual networks are common functional brain characteristics of schizophrenia and depression (Wu et al., 2017). Wolf *et al*. found that after electroconvulsive therapy (ECT), the defense of structural brain networks in patients with depression and schizophrenia, especially the middle temporal gyrus, including the hippocampus and parahippocampal gyrus, was related to the relief of depressive symptoms in patients with depression and psychotic symptoms in patients with schizophrenia (Thomann et al., 2017). In addition, the abnormal density of functional connections in the prefrontal lobe between depression and schizophrenia was a common feature between the two diseases (Han et al., 2019). Using the Granger causality model technique, abnormal structural connections between the prefrontal lobe, thalamus, and cerebellum were found to be a common feature of depression and schizophrenia (Dong et al., 2018). Two studies have reported WM damage in patients with depression, schizophrenia, and bipolar disorder (Dong et al., 2018; Koshiyama et al., 2019). In 2018, it was observed that abnormalities of regional functional connectivity, primary in the bilateral orbitofrontal lobe, are a common brain characteristic of schizophrenia, depression, and bipolar disorder (Wei et al., 2017).

Reduced functional connectivity between the amygdala and dorsolateral prefrontal lobe of adolescents with schizophrenia and depression is a common change in conditions paralimbic GM volume atrophy is a common brain alteration found in patients with depression, bipolar disorder, and schizophrenia (Chang et al., 2018). Recent studies have shown that schizophrenia, depression, and bipolar disorder have the following common characteristic alterations: (a) decreased modularization of the whole brain; (b) decreased activity of frontal–parietal network, subcortical network, and visual network, along with decreased modularization of the sensorimotor network; and (c) the increased connection coefficients between certain networks, especially networks of the dorsolateral prefrontal lobe, angular gyrus, and thalamus (Ma et al., 2019). Sambataro *et al*. previously observed large‐scale network changes in patients with refractory depression and schizophrenia after ECT, with similar differences between the striatal network and default mode network. ECT can improve the connectivity between the large‐scale networks mentioned above to alleviate symptoms in refractory depression and schizophrenia (Sambataro et al., 2019).

It is believed that the whole brain FCD is impaired in first‐episode schizophrenia patients with comorbid depression (Qi et al., 2020). However, there are few studies on this topic. The combination of antipsychotics and antidepressants cannot correct functional alterations in the cerebral cortex in mouse models of schizophrenia with depressive symptoms (Zhou et al., 2020), suggesting that depressive symptoms and schizophrenia symptoms may have additive or synergistic worsening effects on brain function and structure. A few studies have assessed the specific brain signatures of depressive symptoms in schizophrenia by functional magnetic resonance imaging (fMRI). The studies have focused on the “shared neural loop” between schizophrenia and depression. Based on existing symptomatic and epidemiological studies, schizophrenia patients with depressive symptoms have weaker responses to current medications and worse long‐term prognoses than those without depressive symptoms. Additional studies are needed to characterize the brain imaging signatures of schizophrenia patients with depressive symptoms to identify potential therapeutic targets.

### 3.3 Co‐occurring AVHs and depressive symptoms in schizophrenia

Until now, information on the brain imaging characteristics of schizophrenia patients with concurrent AVHs and depressive symptoms has been scarce. Most recently, Siddi *et al*. found that reduced GMV in the prefrontal lobe was associated with depressive symptoms and AVHs in schizophrenia patients (Siddi et al., 2019), and that the severity of AVHs and depressive symptoms was correlated with brain impairments. In our study, significant GMV reductions were detected in the Broca area, Wernicke area, insular lobe, and prefrontal GM in patients with first‐episode schizophrenia with concurrent AVHs and depressive symptoms (Zhuo, Xu, et al., 2020). In addition, the loss of GMV in schizophrenia patients with concurrent AVHs and depressive symptoms was significantly more severe than those patients with depressive symptoms alone (Zhuo, Xu, et al., 2020). Hence, these previous studies, including ours, suggest that depressive symptoms and AVHs may deteriorate reciprocally in patients with first‐episode schizophrenia. Future studies need to assess the precise relationship between the severity degrees of AVHs/depression and brain impairment.

### 3.4 Implications for novel therapeutic strategies to alleviate the reciprocal deterioration of AVHs and depressive symptoms in schizophrenia

As discussed above, abnormalities in some specific regions of the brain are related to the concurrent presence of AVHs and depressive symptoms in schizophrenia, which may help develop therapeutic strategies through normalizing the symptoms‐related brain activity.

Over the past few years, several treatments for schizophrenia patients with concurrent AVHs and depressive symptoms have been explored, such as antipsychotic medications, brain stimulation, and cognitive‐behavioral therapy. However, the efficacy of conventional medications is relatively low. Interestingly, repetitive transcranial magnetic stimulation (rTMS) and transcranial direct current stimulation (tDCS) have emerged as highly efficacious therapies that can normalize the brain regions associated with the depressive and AVH symptoms, as evidenced in functional and structural brain imaging studies. For example, Wang *et al*. modified and applied continuous theta‐burst stimulation (cTBS) to alleviate AVHs in patients with schizophrenia. They found that cTBS could effectively alleviate AVH symptoms in patients by reducing the intensity of FC in the left cerebellum (Chen et al., 2019). In another study, Mondino *et al*. demonstrated that tDCS could relieve auditory hallucinations by reducing the FC between the left temporoparietal junction and anterior left angular gyrus (Mondino et al., 2016). Recently, Zweerings *et al*. used real‐time magnetic resonance imaging (rtfMRI) to show that neural feedback therapy can enhance the neural connections between the language network and default mode network, effectively alleviating the symptoms of AVHs (Zweerings et al., 2019). Similarly, rTMS has been shown to improve the functioning of language‐related networks and internal language networks (Bais et al., 2017), leading to the alleviation of AVH symptoms (Briend et al., 2017).

Audio‐visual assisted therapy aid for refractory auditory hallucinations (AVATAR) is another therapy that has become an effective treatment for AVHs in schizophrenia (Dellazizzo et al., 2018). To date, the relationship between AVATAR and neuroimaging has not been reported. However, in 2019, *Lancet Psychiatry* described the importance of strengthening longitudinal research in this field (Craig et al., 2018). While substantial progress has been made in characterizing the brain imaging targets used in the development of therapeutic strategies for AVHs, there is a lack of longitudinal data to verify these targets. In this context, it is important to identify brain imaging signatures of schizophrenia with concurrent AVHs and depressive symptoms. This will help develop comprehensive treatment strategies, such as antipsychotic and brain stimulation therapies (e.g., neural feedback techniques, rTMS, tDCS, AVATAR), to normalize the brain alterations associated with AVHs and depressive symptoms (Bohlken et al., 2017). Hence, in agreement with previous studies, we have shown that functional alterations in first‐episode schizophrenia patients with AVHs have an “inverted U‐shaped” pattern, in which network connectivity is initially increased, followed by a reduction in connectivity (Zhuo, Xiao, et al., 2020).

### 3.5 Future directions in the treatment of AVHs and depression in patients with schizophrenia

Gray matter volume reductions are associated with the duration and progression of schizophrenia, especially in schizophrenia patients with depressive symptoms or AVHs. Depression has been shown to significantly reduce GM volumes in the prefrontal cortex, particularly in the medial orbitofrontal, right medial superior frontal, superior frontal, and middle frontal gyrus (Raquel et al., 2000; Sara et al., 2018). In addition, Gur *et al*. recently identified a positive correlation between reduced GM orbitofrontal volume and the severity of depressive symptoms in females with schizophrenia (Gur et al., 2000). Since the orbitofrontal cortex plays an essential role in the interaction between self‐monitoring or social behavior and emotional valence, the adverse effect of depressive episodes on the GM orbitofrontal volume may contribute to the self‐monitoring disturbances, which may result in AVHs (Beer et al., 2006; Waters et al., 2012). These findings and those from longitudinal studies suggest that depressive symptoms during the early stages of schizophrenia may exert a causal role in triggering the development of AVHs. This supports the notion that tailored interventions or different treatments may be necessary based on the stage of schizophrenia. The progressive nature of schizophrenia supports the idea early treatment may play a critical role in patients with first‐episode schizophrenia. As first‐episode schizophrenia is often accompanied by depressive symptoms, antidepressants and brain stimulation therapies may help attenuate the neurostructural and functional alterations associated with the disease, possibly preventing the progression of AVHs. These findings also highlight the importance of alternative treatments, such as brain stimulation therapy, by targeting common brain areas (e.g., orbitofrontal cortex) to prevent the reciprocal deterioration of depressive symptoms and AVHs in schizophrenia patients. Further studies are needed to better understand the shared neural mechanisms underlying the reciprocal deterioration of depressive symptoms and AVHs in schizophrenia.

As demonstrated in Table 1, most of the aforementioned studies suggest that depressive symptoms at the early stages of schizophrenia may exert a causal role in triggering the development of AVHs. In view of an essential role of orbitofrontal cortex in the interaction between self‐monitoring or social behavior and emotional valence, the adverse effect of depressive episodes on the GM orbitofrontal volume may contribute to the self‐monitoring disturbances, which may result in AVHs of the orbitofrontal, right medial superior frontal, superior frontal, and middle frontal gyrus. As such, normalization of the functional disruption of orbitofrontal cortex and orbitofrontal cortex‐related networks may alleviate AVHs and depressive symptoms in some patients with schizophrenia.

More notably, in future studies, we must consider the ideal assessment tools to ensure the precise evaluation of symptoms. The Calgary Depression Scale for Schizophrenia (CDSS) is an ideal tool for assessing depressive symptoms in patients with schizophrenia (Addington et al., 1996; Upthegrove et al., 2017). However, CDSS has not been widely used, while only a small proportion of previous studies adopted CDSS to assess depressive symptoms in patients with schizophrenia. Simultaneously, the auditory hallucinations rating scale (Wahab et al., 2015) is considered as an ideal tool for the assessment of AVHs in patients with schizophrenia. As such, these tools are highly recommended to uniform the use of assessment tools for evaluation of the symptoms in future studies aimed at developing tailored treatment strategies.

## 4 CONCLUSION

In conclusion, AVHs and depressive symptoms co‐occur in a proportion of schizophrenia patients, and the reciprocal deterioration may lead to further aggravation of brain damage and decline of social functioning, increasing the overall disability and mortality rates of these patients. Research on the brain imaging signatures of AVHs and depressive symptoms in patients with schizophrenia is still in its infancy. Considering the challenges in treating schizophrenia patients with concurrent AVHs and depressive symptoms, there is an urgent need for in‐depth neuronal mechanistic studies to identify targets for the development of effective therapies to alleviate the reciprocal deterioration.

## CONFLICT OF INTEREST

None declared.

## AUTHOR CONTRIBUTIONS

All the authors listed have made a substantial, direct, and intellectual contribution to the work, and approved it for publication.
